# Inducible Nucleosome Depletion at OREBP-Binding-Sites by Hypertonic Stress

**DOI:** 10.1371/journal.pone.0008435

**Published:** 2009-12-24

**Authors:** Edith H. Y. Tong, Jin-Jun Guo, Song-Xiao Xu, Keri Mak, Sookja K. Chung, Stephen S. M. Chung, Ali-Long Huang, Ben C. B. Ko

**Affiliations:** 1 Department of Anatomical and Cellular Pathology, The Chinese University of Hong Kong, Hong Kong, China; 2 State Key Laboratory in Oncology in South China, The Chinese University of Hong Kong, Hong Kong, China; 3 Key Laboratory of Molecular Biology on Infectious Diseases, Ministry of Education, Institute of Viral Hepatitis, Chongqing University of Medical Sciences, Chong Qing, China; 4 Department of Anatomy, The University of Hong Kong, Hong Kong, China; 5 Department of Physiology, The University of Hong Kong, Hong Kong, China; University of Hong Kong, Hong Kong

## Abstract

**Background:**

Osmotic Response Element-Binding Protein (OREBP), also known as TonEBP or NFAT5, is a unique transcription factor. It is hitherto the only known mammalian transcription factor that regulates hypertonic stress-induced gene transcription. In addition, unlike other monomeric members of the NFAT family, OREBP exists as a homodimer and it is the only transcription factor known to bind naked DNA targets by complete encirclement *in vitro*. Nevertheless, how OREBP interacts with target DNA, also known as ORE/TonE, and how it elicits gene transcription *in vivo*, remains unknown.

**Methodology:**

Using hypertonic induction of the aldose reductase (AR) gene activation as a model, we showed that OREs contained dynamic nucleosomes. Hypertonic stress induced a rapid and reversible loss of nucleosome(s) around the OREs. The loss of nucleosome(s) was found to be initiated by an OREBP-independent mechanism, but was significantly potentiated in the presence of OREBP. Furthermore, hypertonic induction of AR gene was associated with an OREBP-dependent hyperacetylation of histones that spanned the 5′ upstream sequences and at least some exons of the gene. Nevertheless, nucleosome loss was not regulated by the acetylation status of histone.

**Significance:**

Our findings offer novel insights into the mechanism of OREBP-dependent transcriptional regulation and provide a basis for understanding how histone eviction and transcription factor recruitment are coupled.

## Introduction

Exposure of mammalian cells to hypertonic stress induces osmotically obliged water efflux that results in cell shrinkage. In response to the change in volume, membrane transporters have to be activated rapidly to offset volume reduction through increased cellular uptake of electrolytes [1]. However, chronic increase in intracellular ionic strength may inhibit protein synthesis and induce growth arrest [2]. Such deleterious conditions are counteracted by the gradual replacement of electrolytes with organic osmolytes including sorbitol, betaine, myo-inositol, taurine and glycerophosphocholine. These osmolytes replace intracellular electrolytes and preserve cell volume and osmolality without perturbing macromolecular structure and function [3], [4]. The accumulation of organic osmolytes is brought about by the induction of a battery of osmoprotective genes, including aldose reductase (*AR*), betaine/γ-aminobutyric acid transporter (*BGT-1*), and Na^+^-dependent myoinositol transporter (*SMIT*). The hypertonic induction of these genes is controlled at the transcriptional level and is mediated by a common cis-acting element known as the ORE (Osmotic Response Element) or the TonE (tonicity-responsive enhancer) [5], [6], [7], [8].

The transcription factor that binds to the ORE/TonE is known as the Osmotic Response Element-binding Protein (OREBP) or Tonicity-Responsive Enhancer Binding Protein (TonEBP) [9], [10]. OREBP has been shown to be identical in sequence with NFAT5, which was independently identified as a member of the NFAT family of transcription factors [11]. However, OREBP lacks the calcineurin binding domain that is present in other NFAT family members and therefore is regarded as a distant relative [11]. Moreover, OREBP exists as homodimer that binds to its target site by complete encirclement [12]. It is the key transcription factor in the osmoprotective transcription program, and is indispensable in the inner renal medullary cells that are constantly exposed to extreme hypertonicity [13], [14], [15]. Apart from its role in osmoadaptation in renal tubular cells, OREBP is also widely expressed in other mammalian cell types that are not of renal origin [10], [16], [17], [18], [19], and has been implicated in cellular differentiation [20], [21], [22], cancer metastasis [23], and drug metabolism [24].

Earlier studies have established that extracellular tonicity regulates OREBP through altering its subcellular localization. Hypotonicity induces nuclear export of OREBP, mediated by Casein Kinase 1-dependent phosphorylation [25], whereas hypertonic stress induces rapid translocation of OREBP from the cytoplasm to the nucleus [26]. Furthermore, hypertonicity also phosphorylates and activates the transcriptional activity of OREBP associated with the activity of ataxia telangiectasis-mutated kinase (ATM) [27], Protein Kinase A [28], and Casein Kinase 2 [29], although the precise signaling pathway leading to its activation remains largely undefined.

To carry out gene transcription, transcription factors must first gain access to their cognate DNA sequences for direct interaction. However, eukaryotic DNA is wound around histone octamers to form nucleosomes, and the nucleosomes are further packaged into highly compacted chromatin structures. Consistent with these observations, emerging evidence has suggested that chromatin also plays an active role in the regulation of gene expression. Inducible gene transcriptions are associated with an alteration in chromatin structure, which renders the DNA accessible to the transcription factors [30]. This could be brought about by covalent modifications of histone tails that weaken the nucleosome-DNA interaction [31], or by histone remodeling enzymes that disassemble the nucleosomes or displace nucleosomes along the chromatin [32], [33].

Earlier studies have focused on the mechanism of hypertonicity-induced nuclear translocation and activation of OREBP [26], [27], [28], [34], [35]. On the other hand, the mechanism of how OREBP is recruited to the target sites remains largely unexplored. Because hypertonic stress leads to covalent modifications of histones [36], [37], we hypothesized that the histone modifications or remodeling might directly impinge on OREBP-mediated gene transcription by regulating its accessibility to target DNA. Many of the currently known OREBP-regulated genes including AR [5], SMIT [8], and HSP70 [6] contain multiple OREs/TonEs that are located far from the proximal promoter regions, which allow us to discern the changes in chromatin structure at the ORE sites specifically. Herein we examined the role of nucleosomes in OREBP-mediated gene transcription. By studying hypertonic induction of the AR gene, we show that hypertonic stress induced eviction of one or more nucleosomes specifically at the genomic region containing the OREs. Interestingly, while nucleosome eviction could be initiated in the absence of OREBP, maximal nucleosome eviction was observed only in the presence of the transcription factor. Furthermore, we also observed that hypertonic stress induced histone acetylation that spanned the 5′ upstream sequences and the exons of the AR gene in an OREBP-dependent manner, implicating that histone acetylation has a role in AR gene activation.

## Materials and Methods

### Cell Cultures

HeLa cells (American Type Culture Collection) were cultured in Minimum Essential Medium supplemented with 10% fetal bovine serum, glutamine, sodium pyruvate, penicillin and streptomycin. NIH-3T3 cells (American Type Culture Collection) were cultured in Dulbecco's modified Eagle's medium containing 10% fetal bovine serum, glutamine, penicillin and streptomycin. To obtain mouse embryonic fibroblasts (MEFs), mice heterozygous for the OREBP alleles (OREBP^+/−^) on a SVJ129/C57BL/6N background were crossbred [38]. MEFs that are wild type (OREBP^+/+^), or null (OREBP^−/−^) for OREBP were derived from day 14.5 embryos as described [39]. MEFs were cultured in Dulbecco's modified Eagle's medium supplemented with 10% fetal bovine serum, glutamine, penicillin, and streptomycin. The experiments involving animals were carried out in accordance with the guidelines of the Committee on the Use of Live Animals in Teaching and Research, the University of Hong Kong. Expression of OREBP in mouse embryonic fibroblasts was confirmed by Western blotting analysis using anti-OREBP antibodies. All cells were maintained in a humidified incubator at 37°C with 5% CO_2_. All reagents for cell cultures were purchased from Invitrogen. Hypertonic medium (550 mosmol/kg H_2_O) was prepared by supplementing NaCl solution to the growth medium. Medium osmolality was measured by the Vapro® vapor pressure osmometer (Wescor).

### Antibodies

Antibodies against histone H3, H4 and H2B, acetyl-histone H3 (H3AcK9K14) and acetyl-histone H4 (H4AcK3K8K12K16) were purchased from Upstate Biotechnology. Polyclonal OREBP antibody was raised in rabbits using recombinant His-tagged OREBP protein (corresponds to amino acid residues 78–543 of human OREBP) and anti-OREBP IgG was subsequently purified using affinity chromatography.

### Quantitative Reverse Transcription PCR (qRT-PCR)

First-strand cDNA was synthesized from 1 µg of total RNA using High Capacity RNA-to-cDNA Master Mix (Applied Biosystems) according to manufacturer's instructions. Genomic DNA was digested by DNase I (New England Biolabs). Quantitative PCR experiments were performed using the SYBR Green PCR Core reagent kit (Applied Biosystems) and the reactions were carried out using an ABI 7900 real-time PCR system (Applied Biosystems). The expression analysis was performed in triplicate using TaqMan Gene Expression Assays for mouse aldose reductase and normalized to β-actin (Applied Biosystems). The ΔΔCt method of relative quantification was used to compare the level of expression in different treatments.

### Chromatin Immunoprecipitation (ChIP) Assays and Real Time PCR

The ChIP assay was performed as described [40] with slight modifications. Unstimulated or hypertonicity-induced MEFs, NIH-3T3 cells or HeLa cells (3×10^7^) were crosslinked for 15 min at 4°C using 1% formaldehyde. Cells were harvested and resuspended in 400 µl cell lysis buffer (50 mM Tris-Cl pH 8.0, 10 mM EDTA and 1% SDS, 1 mM PMSF and 1X protease inhibitor) and were kept on ice for 10 min. Cell lysates were sonicated by Ultrasonic Processor VCX400 (Sonics & Materials Vibra-cell) in an ice bath to produce DNA fragments ranging from 200–1000 bp as determined by agarose gel electrophoresis. After the removal of debris by centrifugation, the lysates were diluted 10-fold with ChIP dilution buffer (16.7 mM Tris-Cl pH 8.1, 2 mM EDTA pH 8.0, 167 mM NaCl , 0.01% SDS and 1.1% Triton X-100), and pre-cleared with Protein A Agarose and Salmon sperm DNA (Upstate Biotechnology) at 4°C for 30 min. Chromatin immunoprecipitation was carried out using anti-histone (H3, H4, H2B or acetyl-H4) or anti-OREBP antibodies. The antibody-chromatin complexes were then precipitated by Protein A Agarose/Salmon sperm DNA, washed and eluted. DNA and proteins were reverse crosslinked, and digested with protease K. The eluted DNA was purified by QIAquick spin column (Qiagen). For the analysis of the AR gene, input and immunoprecipitated DNA was analyzed by quantitative PCR using primers and Taqman probes (Applied Biosystems). Quantitative PCR was conducted using an ABI 7900 real-time PCR system (Applied Biosystems). Each sample was performed in triplicate. To quantify the amount of DNA, a standard curve was generated from serial dilutions of a known amount of genomic DNA. The absolute amount of ChIP and input DNA was calculated by correlating the standard curve. The amount of immunoprecipitated target sequence was calculated by normalization with the total input DNA. For the analysis of the TonEA region of the human SMIT gene, DNA was quantified by an SYBR Green PCR Core reagents kit (Applied Biosystems). The sequences of primers and Taqman probes used for the assay of the AR gene were as follows: AR intergenic region (AR_INTER,_ −3515 bp to −3420bp): Forward: 5′–CACTTTGGCTCTCTTGGGACAA–3′, Probe: 5′–TCCAACCCAAATCTTC–3′, Reverse: 5′– ACTCAGGGTACAGAGGTTTCCT–3; OREs region (AR_ORE_, -1182 bp to -1023 bp): Forward: 5′–GGGTGGGAGTGGAGAAGTG – 3′, Probe: 5′–CCGACTGGAAAATC–3′, Reverse: 5′–CCCACCTCTCTAAATCCCATTCTG–3′; Proximal promoter region (AR_PP_, -30 bp to +102 bp): Forward: 5′–CAATCAGAAGGATCCGTCTCTCA–3′, Probe: 5′–CCTGCCATTGGTTGCAC–3′, Reverse: 5′–CCCAACGGCCTGTAGAAAGAA–3′; and Exon 2 (AR_EX2_, +4565 bp to +4732 bp): Forward: 5′–TGGCTTATACTCCTCCCTTTCCA–3′; Probe: 5′–CGGTACCCCAAGTCAATAGCA–3′, Reverse: 5′–ACTGAGGCCGTGAAAGT–3′). The sequences of primers used for the assay of SMIT gene were: forward: 5′-GCTGTAGCCA CTCCCTTTGC-3′, reverse: 5′-GGAGGCTGGTCTTGGACTCT-3′.

### Micrococcal Nuclease (MNase) Digestion and Southern Blotting Analysis

The MNase assay was carried out as described with slight modifications [41]. In brief, cells were collected by centrifugation at 4°C and cell pellets were resuspended in lysis buffer (10 mM Tris-HCl, pH 7.4 10 mM NaCl, 3 mM MgCl_2_, 0.5% NP-40, 0.15 mM spermine, 0.5 mM spermidine). Cell nuclei were collected by centrifugation and washed once with MNase digestion buffer (10 mM Tris-HCl, pH 7.4, 15 mM NaCl, 60 mM KCl, 0.15 mM spermine, 0.5 mM spermidine). The nuclei were incubated with 40U MNase (Fermemtas) at 20°C. Samples were removed at 4, 10, 16, 22, and 28 min intervals. The digestion was stopped by the addition of MNase stop buffer (20% MNase digestion buffer, 100 mM EDTA and 10 mM EGTA pH 7.5). The mixture was subjected to overnight digestion in proteinase K in the presence of 1% SDS. Genomic DNA was purified by phenol-chloroform DNA extraction. 20 µg of the purified DNA was resolved using 1.3% agarose-TBE gel, transferred to a nylon membrane (Whatman), and hybridized to a 153 bp-DNA fragment that spanned the OREs (ORE_MNase_, –1155 bp to –1003 bp) region. Probe labeling was performed using Random Prime Labeling (Amersham Biosciences) in the presence of ^32^P-dCTP. Radioactive images were captured by X-ray film.

## Results

### Apparent Reduction of Histone Acetylation at AR OREs in Response to Hypertonic Stress

**Figure 1:**
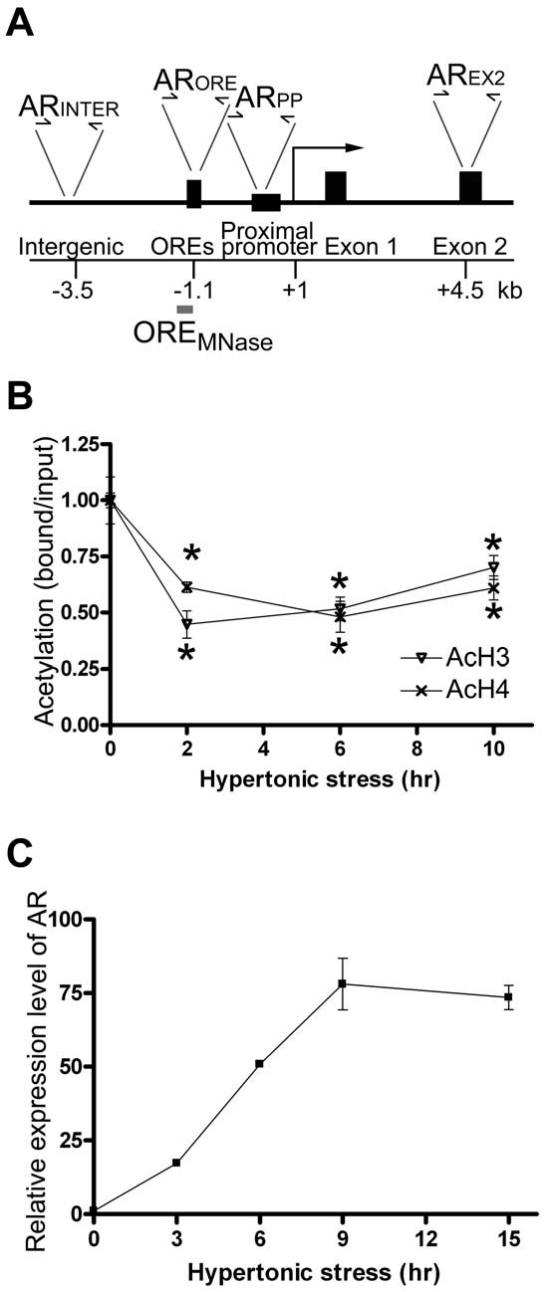
Evaluation of histone acetylation status at the OREs of AR in response to hypertonicity. (A) Schematic illustration of the mouse AR genomic DNA showing exon 1 and 2, promoter and upstream region. The region amplified by primer sets AR_INTER_, AR_ORE_, AR_PP_ and AR_EX2_ are indicated by arrows. Transcriptional start site is indicated by a bold arrow. The DNA probe for southern blotting analysis in the MNase digestion assay (ORE_MNase_) is indicated by a grey bar. (B) ChIP assays of AR promoter. ChIP assays were performed with antibodies against acetylated histone H3 or acetylated histone H4 using NIH-3T3 cells that were induced with hypertonicity for 0, 2, 6 and 10 h, respectively. DNA was analyzed by real-time PCR using AR_ORE_ primer pair. The results are normalized to the ratio of immunoprecipitated DNA to total input DNA at time 0. Data are the mean ± SEM (n = 3). *, p<0.05 by one-way ANOVA test when compared to the value at time 0. (C) Quantitative analysis of AR transcriptions by RT-PCR. NIH-3T3 cells were incubated in hypertonic medium for 0, 3, 6, 8 and 15 h. Total RNA was prepared. Relative expression level of AR was determined. The expression level of β-actin was used to normalize the AR expression. Results were expressed as fold change relative to the expression level at time 0. Data are the mean ± SEM (n = 3).

Three functional OREs which covers ∼90 nucleotides and are arranged in tandem, are located 1.1 kb upstream of the *AR* promoter [5] and interact with OREBP *in vivo* [17]. As histone modifications regulate the accessibility of chromatin and gene activity [42], [43], [44], we first examined if hypertonicity induces, in vicinity of the OREs, histone acetylation, which is known to regulate chromatin folding [45] and is a hallmark of gene activation [46], [47], [48]. We carried out chromatin immunoprecipitation (ChIP) followed by quantitative PCR amplification of a 160 bp amplicon that spanned the OREs (Fig. 1A, AR_ORE_). Unexpectedly, the ChIP signal for acetylated histone H3 and H4 at AR_ORE_ was reduced significantly upon hypertonic treatment in a time-dependent manner. The reductions were observed as early as at 30 min and were the lowest for the AcH4 antibody at 2 hr and the AcH3 antibody at 6 hours respectively (Fig. 1B). In contrast, *AR* was actively transcribed throughout this period (Fig. 1C).

### Hypertonic Stress Induced Nucleosome Depletion at the OREs

**Figure 2:**
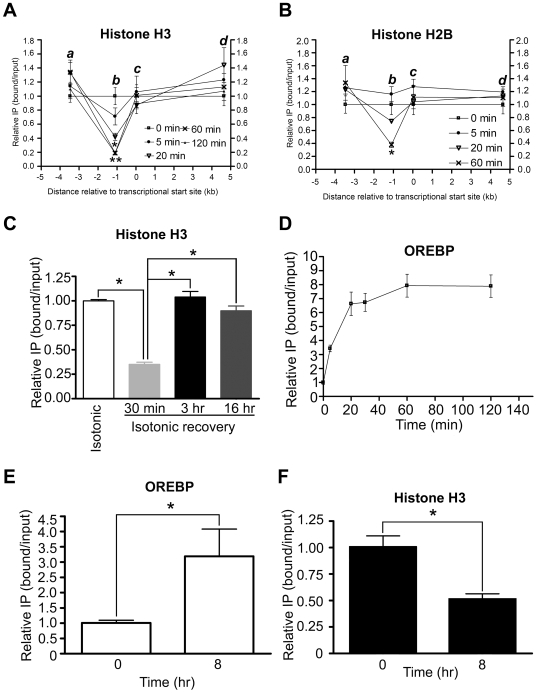
Chromatin changes at the AR gene in response to hypertonic stress. (A-C) NIH-3T3 cells were subjected to hypertonic stress, and ChIP was conducted with antibodies against (A) histone H3, (B) histone H2B, and (C) histone H3. DNA was analyzed by real-time PCR using AR_INTER_ (a), AR_ORE_ (b), AR_PP_ (c), and AR_EX2_ (d) primer pair respectively as indicated in Figure 1. *, p<0.01 and **, p<0.001 by one-way ANOVA. (C) Cells were either left untreated (isotonic), or treated with hypertonic medium for 30 min followed by incubation in isotonic medium for 30 min, 6 and 16 h respectively (isotonic recovery). ChIP was conducted with antibody against histone H3. DNA was analyzed by real-time PCR using AR_ORE_ primer pair. The results are presented as the ratio of immunoprecipitated DNA to total input DNA and normalized to the value of cells maintained under isotonicity. *, p<0.001 by one-way ANOVA. (D) Cells were subjected to hypertonic stress, and ChIP was conducted with antibodies against OREBP. DNA was analyzed by real-time PCR using AR_ORE_. The results were normalized to the ratio of immunoprecipitated DNA to total input DNA at time  = 0. (E and F) Cells were treated with hypertonicity for 0 and 8 hr, respectively. ChIP was conducted with antibodies against OREBP (E) or histone H3 (F). DNA was analyzed by real-time PCR using a primer pair specific for the TonEA of the SMIT gene. The results were normalized to the ratio of immunoprecipitated DNA to total input DNA at time  = 0. *, p<0.05 by Student's t-test. For A-G, data are the mean ± SEM (n = 3).

In yeast, active regulatory elements and promoters are usually nucleosome depleted [48], [49]. It is therefore plausible that the observed reduction in the levels of acetylated histone at the OREBP-binding sites might have resulted from nucleosome depletion *per se* in response to hypertonic induction. We hence analyzed the chromatin structure over a region of 8 kb encompassing regions upstream and downstream of the *AR* promoter. A nucleosome consists of approximately 146 bp of DNA wrapped around an octamer of histone proteins (two each of H2A, H2B, H3 and H4). We first examined histone H3 occupancy after hypertonic stress by ChIP followed by quantitative PCR amplification of four amplicons that, in addition to the AR_ORE_, spread across a 8 kb region of the gene, including a -3.5 kb intergenic region (AR_INTER_), a proximal promoter (AR_PP_), and an exon 2 (gene body) region (AR_EX2_) respectively (Fig. 1A). As shown in Fig. 2A, we observed the depletion of histone H3 specifically at the locus of the OREs, whereas histone integrity at the other three loci was not altered significantly by hypertonic stress. Histone loss was observed at 5 min after hypertonic treatment and was reduced maximally by five-fold at 60 min. Hypertonic treatment for 120 min did not lead to further reduction of histone H3. Similarly, the quantities of histone H2B (Fig. 2B) and histone H4 (data not shown) at the same locus were reduced at a rate and magnitude comparable to that of histone H3, suggesting that whole nucleosome located over the ORE region of *AR* was depleted in response to hypertonic stress. Notably, the level of histone H3 could be brought back to normal level when cells were returned to isotonic conditions (Fig. 2C), suggesting that nucleosome integrity could be restored upon removal of stress. On the other hand, when ChIP was conducted using anti-OREBP antibody, we showed that OREBP was rapidly recruited to the ORE *in vivo* in response to hypertonic stress (Fig. 2D). The kinetics of OREBP recruitment was inversely correlated with histone depletion (compare Fig. 2A and Fig. 2D), which would suggest that these two events are coupled. To elucidate if histone loss at the ORE is a general phenomenon during the activation of other OREBP-dependent genes, we analyzed histone H3 occupancy at another putative ORE loci (TonEA) located upstream of the human SMIT gene promoter [8]. Our data confirmed that TonEA interacts with OREBP *in vivo* (Fig. 2E). Furthermore, similar to *AR*, histone H3 was also depleted from TonEA upon hypertonic stress (Fig. 2F). Taken together, our data suggest that histone depletion at the ORE was associated with OREBP-dependent gene activation.

**Figure 3:**
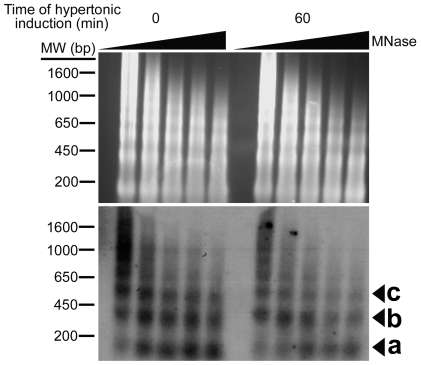
Increased MNase accessibility at the ORE region after hypertonic induction. (upper) Nuclei were prepared from cells that were induced with hypertonic stress for 0 and 60 min respectively, and chromatin was subjected to limited digestion with MNase increasing times. The nucleosome ladder was resolved in agarose gel and visualized by ethidium bromide staining; (lower), the DNA was subjected to Southern blots analysis using a ^32^P-labeled probe spanning the ORE (ORE_MNase_, Fig. 1A). The triangles denote increasing times of MNase digestion. a, mononucleosome; b, dinucleosomes; c, trinucleosomes.

To confirm that the observed reduction in ChIP signals was not due to epitope inaccessibility of the antibodies by large protein complexes in the chromatin, we analyzed the sensitivity of *AR* chromatin to micrococcal nuclease (MNase) digestion [50]. The susceptibility of chromatin to MNase had been used to identify nucleosomal and linker DNA previously [51]. Isolated nuclei from NIH-3T3 fibroblasts subjected to limiting digestion with MNase produce a ladder of DNA fragments corresponding to multiples of the nucleosome (Fig. 3, upper). This was followed by southern blotting analysis using a 153 bp probe that spans only the three OREs (ORE_MNase_, Fig. 1A). We observed a clear nucleosome ladder when cells were maintained at the resting state. A nucleosome ladder corresponding to one to four nucleosomes could be clearly distinguished, whereas at five nucleosomes or more it became nebulous. In response to hypertonic stress, the intensities of the MNase-protected bands was diminished, suggesting that there was a reduction in nucleosome density (Fig. 3, lower). Since the reduction in the intensities of MNase-protected bands paralleled the loss of histones as determined by ChIP-qPCR analysis, we concluded that nucleosomes undergo eviction in vicinity of OREs in response to hypertonic stress.

### OREBP-Dependent H4 Histone Acetylation at AR OREs in Response to Hypertonic Stress

**Figure 4:**
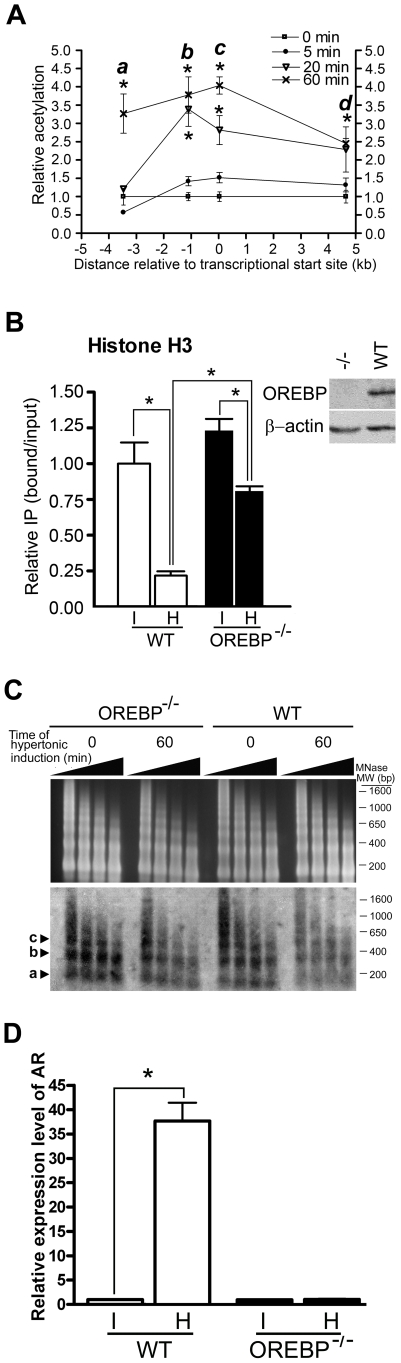
Histone acetylations and histone occupancy at the AR gene in response to hypertonicity. (A) Histone H4 hyperacetylation at the AR gene in response to hypertonic stress. Cells were induced with hypertonic stress, and ChIP was conducted with antibodies against acetylated histone H4 and histone H4. DNA was analyzed by real-time PCR using AR_INTER_ (a), AR_ORE_ (b), AR_PP_ (c), and AR_EX2_ (d) primer pair respectively. The ratio of immunoprecipitated DNA to total input DNA for each of the antibodies was determined. The value for the level of acetylation of histone H4 was divided by H4 occupancy value. Values at time 0 were set to be 1.0. *, p<0.01 by one-way ANOVA. (B) Histone occupancy at the ORE region of WT and OREBP^−/−^ MEFs. Cells were maintained under isotonicity or treated with hypertonicity for 1 hr. ChIP was conducted with antibody against histone H3. DNA was analyzed by real-time PCR using AR_ORE_ primer pair. The results are presented as the ratio of immunoprecipitated DNA to total input DNA and normalized to the value of WT MEFs maintained under isotonicity. *, p<0.01 and **, p<0.001 by one-way ANOVA. I, isotonic medium; H, hypertonic medium. Insert, Western blotting analysis of MEFs using anti-OREPB antibodies. (C) MNase assay. (Upper) Nuclei were prepared from WT and OREBP^−/−^ cells that were induced with hypertonic stress for 0 and 60 min, respectively, and chromatin was subjected to limited digestion with MNase for increasing times. The nucleosome ladder was resolved in agarose gel and visualized by ethidium bromide staining; (Lower). The DNA was subjected to southern blot analysis using a ^32^P-labeled probe spanning the ORE (ORE_MNase_, Fig. 1A). The triangles denote increasing times of MNase digestion. a, mononucleosome; b, dinucleosomes; c, trinucleosomes. (D) Quantitative analysis of AR transcriptions by RT-PCR. WT and OREBP-/- fibroblasts were incubated in isotonic or hypertonic medium for 16 h. Total RNA was prepared. Relative expression level of AR was determined. The expression level of β-actin was used to normalize the AR expression. Results were expressed as fold change relative to the expression level at time 0. Data are the mean ± SEM (n = 3).

Because a progressive loss of nucleosomes in the vicinity of the OREs was observed, we reasoned that the measurement of acetylated histone alone (Fig. 1B) might not faithfully reflect the chromatin acetylation status of the AR gene, especially at the ORE locus. We therefore re-examined the amount of acetylated histone of these loci by normalizing the level of acetylated histone H4 to nucleosome density (histone H4). A similar strategy has been used to evaluate the level of acetylated histones in yeast PHO5 promoter in which nucleosomes were evicted upon gene activation [52]. After normalization, we found that, despite a progressive reduction of nucleosomes by hypertonic stress, there was a time-dependent increase in the level of acetylated histone H4 at the ORE locus. Furthermore, there was also an increase in the level of histone H4 acetylation (2.5- to 4-fold) at the other three loci under hypertonicity (AR_INTER_, AR_PP_, AR_EX2_) (Fig. 4A). Collectively, our data suggest that histone H4 hyperacetylation was associated with gene activation.

Because nucleosome disassembly or remodeling can be promoted by its acetylation [52], or by binding of the transcription factor to target sites [53], [54], [55], the correlation between OREBP recruitment, histone acetylation and nucleosome eviction was analyzed. To this end, we analyzed the chromatin structure of the AR gene using mouse embryonic fibroblasts (MEFs) derived from wild-type (WT) and OREBP-deficient (OREBP^−/−^) mice. As shown in Fig. 4B, consistent with our findings using NIH-3T3 cells, ChIP-qPCR analysis of WT and OREBP^−/−^ fibroblasts showed a four-fold reduction in histone H3 occupancy at the ORE locus at 60 min after hypertonic stress. On the other hand, a significant reduction in histone H3 occupancy was also observed in OREBP^−/−^ fibroblasts in response to hypertonic stress, although to a lesser extent when compared to the WT fibroblasts. Hypertonic induction for up to 3 hrs did not lead to further reduction of histone H3 level in OREBP^−/−^ fibroblasts (data not shown), suggesting that the initiation of histone eviction in response to hypertonic stress was carried out in an OREBP-independent manner, but OREBP might further promote nucleosome eviction upon binding to the ORE. In addition, consistent with our postulation that OREBP was not required for nucleosome eviction, MNase analysis of WT and OREBP^−/−^ fibroblasts revealed that hypertonicity reduced the intensity of MNase-protected bands in both cell types, although a more prominent reduction was observed with the WT cells (Fig. 4C). It was unlikely that nucleosome eviction as observed in the OREBP^−/−^ fibroblasts was mediated by functional complementation of other NFATs, because hypertonic induction of *AR* was completely blunted in OREBP-deficient cells (Fig. 4D).

**Figure 5:**
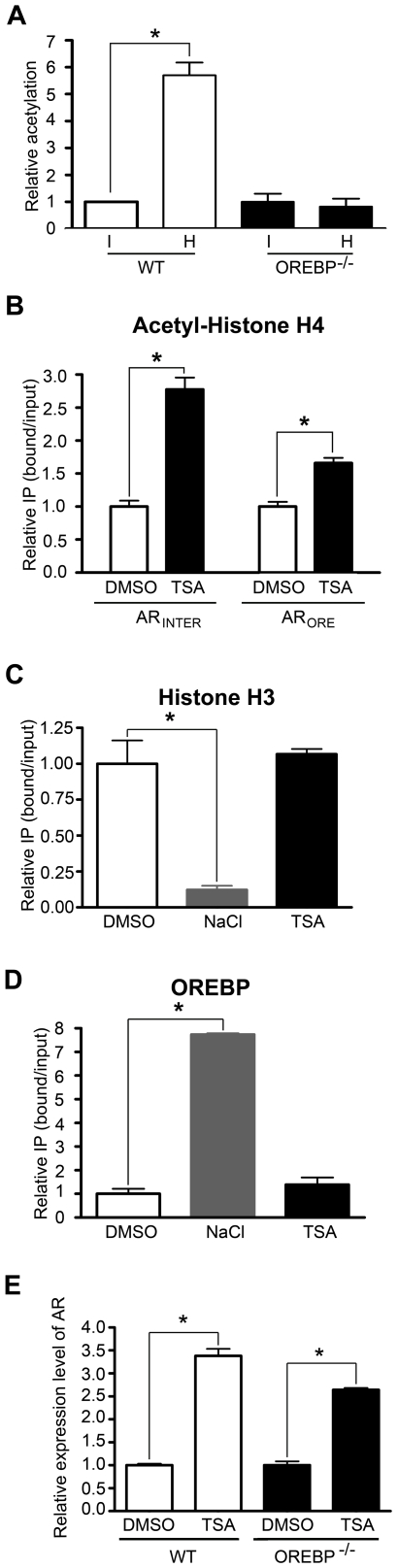
Association between OREBP, histone acetylation and histone eviction. (A) Role of OREBP in histone H4 acetylation. WT and OREBP^−/−^ MEFs were maintained under isotonicity or treated with hypertonic medium for 1 h. ChIP was conducted with antibody against acetylated histone H4 and histone H4, respectively. DNA was analyzed by real-time PCR using AR_ORE_ primer pair. The ratio of immunoprecipitated DNA to total input DNA for each of the antibodies was determined. The value for acetylation histone H4 level was divided by H4 occupancy value. The value of WT MEFs under isotonic conditions was set to be 1.0. *, p<0.001 by one-way ANOVA. I, isotonic medium; H, hypertonic medium. (B-E) Effect of TSA treatment on histone eviction at ORE and AR expression. WT MEFs were treated with DMSO or TSA (1 µg/ml) for 2 hr. ChIP was conducted with antibody against (B) acetylated histone H4, (C) histone H3, and (D) OREBP. In (B), DNA was analyzed by real-time PCR using AR_ORE_ and AR_INTER_ primer pairs. The ratio of immunoprecipitated DNA to total input DNA for each of the antibodies was determined. Values of DMSO treatment were set to be 1.0. *, p<0.05 by one-way ANOVA. In (C) and (D), DNA was analyzed by real-time PCR using AR_ORE_ primer pair. The ratio of immunoprecipitated DNA to total input DNA for each of the antibodies was determined. The results were normalized to the ratio of immunoprecipitated DNA to total input DNA of DMSO treatment. *, p<0.01 by one-way ANOVA. (E) Quantitative analysis of AR transcriptions by RT-PCR. Cells were treated with DMSO or TSA (1 µg/ml) for 8 h. Total RNA was prepared and relative AR mRNA level was determined. The result was expressed as fold change relative to the expression level of WT MEFs treated with DMSO. Data are the mean ± SEM (n = 3). *, p<0.001 by one-way ANOVA.

To determine the role of OREBP in hypertonicity-induced histone hyperacetylation, we measured the level of acetylated histone H4 of *AR* in WT and OREBP^−/−^ fibroblasts respectively. As shown in Fig. 5A, similar to the findings with NIH-3T3 cells, we observed an increase in histone H4 acetylation over the ORE when WT fibroblasts were subjected to hypertonic stress. In contrast, histone H4 acetylation was completely abolished in OREBP^−/−^ fibroblasts, suggesting that OREBP was mandatory for the acetylation of histone H4. To elucidate the role of histone acetylation in nucleosome eviction, we examined if trichostatin A (TSA), an inhibitor of histone deacetylase, promotes nucleosome eviction in the absence of hypertonic stress. In the event, TSA promoted acetylation of histone H4 over the ORE and the intergenic region (Fig. 5B). However, it neither induced nucleosome loss (Fig. 5C) nor enhanced the recruitment of OREBP to ORE (Fig. 5D), suggesting that histone acetylation did not play a role in nucleosome eviction and OREBP recruitment. Nevertheless, TSA significantly increased the levels of *AR* mRNA in WT and OREBP^−/−^ fibroblasts under isotonic conditions (Fig. 5E). The observation was consistent with the notion that gene transcription in general was promoted by histone acetylation [46], [47], [48].

## Discussion

DNA is wrapped around the nucleosome core and is subsequently packed into chromatin fiber. However, the nucleosome is in a dynamic state that can be regulated by post-translational modifications and/or remodeling, which is important for a number of cellular events ranging from gene transcriptions [56], [57] to DNA break repairs [58]. Post-translational modification of histone and nucleosome remodeling may facilitate the recruitment of transcription factors to DNA by acting as a docking signal and weakening of nucleosome-DNA contacts, respectively [59], [60]. Consistent with these notions, many of the active promoters are depleted of nucleosomes [48], [49] or are associated with nucleosome eviction [61]. *In vitro* studies suggested that the binding of transcription factors to nucleosome-DNA complex could induce nucleosome eviction [53], [54], [55]. This model was supported by *in vivo* studies demonstrating that the binding of Pho4 to PHO5 promoter [62], [63], the binding of progesterone receptor to mouse mammary tumor virus promoter [64], as well as the recruitment of NFAT and c-Rel to the proximal promoter of interleukin-2 gene [65], resulted in nucleosome evictions. Contrary to the existing paradigm, our data suggested that hypertonic stress-induced nucleosome eviction at the ORE locus did not require the recruitment of OREBP. Instead, in response to hypertonic stress, nucleosomes at or flanking the ORE sites were evicted before interacting with OREBP. It is possible that the eviction was mediated by some chromatin remodeling factors located in proximity to the ORE, which might response rapidly to the hypertonic signal.

What is the biological significance of the ORE region being associated with dynamic nucleosomes? We speculate that such a regulatory mechanism may have evolved along with the first appearance of OREBP in flies [66]. OREBP is distinctive from other known transcription factors in that the intrinsic affinity of monomeric OREBP to ORE is low, but the stability of transcription factor-DNA complex is greatly enhanced after complete encirclement of DNA by its dimeric from. Importantly, the lumen formed by the OREBP dimer is sufficient to accommodate only naked DNA [12]. Therefore, a tight control on OREBP transcriptional activity could be achieved by using the nucleosome as a regulatory switch. Nucleosomes could impose steric hindrance to OREBP during the resting state. In contrast, the exposure of ORE via nucleosome(s) eviction and the increase in nuclear abundance of OREBP [26] under hypertonic stress might significantly promote the odds of OREBP-ORE interactions. Although our data suggested that the OREs of AR contained positioned nucleosomes, the number of nucleosomes that were subjected to eviction under hypertonic stress remained unknown. The mapping of nucleosome positions at the OREs locus may help to clarify this issue and would be important to further our understanding of the role of chromatin remodeling in hypertonicity-induced gene expressions.

How hypertonic stress directs locus-specific histone eviction remains elusive. Hypertonic stress may either mark these histones by a specific or a combination of post-translation modifications that could be recognized by precise remodeling factors. Alternatively, nucleosomes over ORE may be composed of specific histone variants which render them distinctive. Recently it has been shown that histone variant H2A.Z is preferentially incorporated into transcription factor binding sites [67]. H2A.Z has also been shown to form less stable nucleosomes *in vitro* [68], undergo eviction in response to DNA damage and promote gene transcription *in vivo* [67]. Therefore characterizing the identity and the nature of post-translational modifications of these nucleosomes may provide important clues to the molecular mechanisms of the histone eviction process. On the other hand, it will also be important to characterize the role of known ATPase-containing histone remodeling factors, such as Swi/Snf-remodeling complexes that are known to mediate histone displacement *in vitro* [69], [70], [71], in OREBP-dependent gene transcription. It is becoming apparent that the genome utilizes a combination of DNA sequences and epigenetic modifications to direct active gene transcription or to achieve gene silencing. Our study suggests that chromatin undergoes chromatin remodeling by nucleosome eviction to recruit transcription factors to its target sites for gene activation.
